# Long noncoding RNA UCA1 knockdown inhibits cisplatin-resistant cervical cancer tumorigenesis via the miR-195-5p/IKBKB axis: LncRNA UCA1 promotes cisplatin-resistant cervical cancer progression

**DOI:** 10.3724/abbs.2025032

**Published:** 2025-04-22

**Authors:** Bi Wang, Ling Li, Zhengyu Wu, Xuanzhen Qian, Wenfeng Yu, Zhi Huang

**Affiliations:** 1 Key Laboratory of Endemic and Ethnic Diseases Ministry of Education School of Basic Medical Guizhou Medical University Guiyang 550004 China; 2 Key Laboratory of Molecular Biology School of Basic Medicine Guizhou Medical University Guiyang 550004 China; 3 Department of Interventional Radiology the Affiliated Hospital of Guizhou Medical University Guiyang 550002 China

**Keywords:** long noncoding RNA UCA1, cervical cancer, cisplatin resistance, miR-195-5p, IKBKB, BNIP3

## Abstract

Cisplatin resistance is a major cause of poor prognosis in patients with cervical cancer. Dysregulation of long noncoding RNAs (lncRNAs) plays a key role in chemoresistance. Our results reveal that the lncRNA UCA1 is upregulated in cisplatin (DDP)-resistant cervical cancer tissues and HeLa cells. Mechanistically, the lncRNA UCA1 acts as a sponge for miR-195-5p, targeting IKBKB. UCA1 enhances proliferation, migration, and invasion while reducing apoptosis in DDP-resistant HeLa cells via the miR-195-5p/IKBKB axis. Additionally, UCA1 upregulates BNIP3Δex2 and p-p65 expressions and downregulates BNIP3 expression in DDP-resistant HeLa cells. Abnormal expressions of BNIP3Δex2 and BNIP3 significantly alter the malignant progression of HeLa/DPP cells. *In vivo*, *UCA1* silencing inhibits growth, enhances apoptosis, and upregulates IKBKB, BNIP3Δex2, and p-p65 expressions while downregulating BNIP3 expression in subcutaneous xenografts in nude mice by targeting miR-195-5p. Overall, this study highlights a novel promising target for the treatment of DDP-resistant cervical cancer.

## Introduction

Cervical cancer is the fourth most common cancer among women worldwide [1], with 604,127 cases and 341,831 deaths in 2020 [2]. Treatment primarily includes surgery, radiotherapy, and chemotherapy [3], with chemotherapy being the main clinical strategy for advanced cervical cancer [4]. Cisplatin (DDP), a commonly used chemotherapeutic agent, induces severe DNA damage and cell death [5]. While effective initially, most patients develop DDP resistance, leading to reduced drug efficacy and potential cancer recurrence or metastasis [6]. The prognosis for patients with recurrent or advanced metastatic cervical cancer remains poor, with a 5-year survival rate of 16.5% [7]. Thus, exploring the mechanism of DDP resistance is crucial for improving chemotherapy outcomes.

Long noncoding RNA (lncRNA) are a novel class of noncoding RNAs longer than 200 nucleotides that do not encode proteins [8]. They play significant roles in cancer processes, such as proliferation, metastasis, and apoptosis [ 9– 11]. LncRNA UCA1 (urogenital carcinoma antigen 1) is highly expressed in multiple tumors, including gastric cancer [12], colorectal cancer [13], and breast cancer [14]. UCA1 has been reported to enhance DDP resistance in various cancers, such as gastric cancer [15], lung adenocarcinoma [16], and ovarian cancer [17]. Recent studies have shown that UCA1 accelerates cervical cancer progression by targeting SMARCD3 or miR-145 [ 18, 19]. Therefore, we hypothesized that UCA1 may enhance DDP resistance in cervical cancer. LncRNAs can act as competitive RNAs of miRNAs (ceRNAs) to regulate tumor metastasis and drug resistance [ 20, 21]. Bioinformatics analysis suggested that miR-195-5p targets and binds to *UCA1* and *IKBKB*. However, it remains unclear whether UCA1 induces DDP resistance in cervical cancer cells via the miR-195-5p/IKBKB axis.

IKBKB overexpression activates NF-κB (p65), regulates apoptosis-related genes, and inhibits cell apoptosis [22]. The activation of NF-κB can also cause DDP resistance in cervical cancer cells [23]. These studies suggested that UCA1 might promote NF-κB activation by upregulating IKBKB, leading to DDP resistance in cervical cancer. Furthermore, NF-κB has been shown to decrease BNIP3 expression [24], and BNIP3 can be selectively spliced to produce BNIP3∆ex2, preventing cell apoptosis [25].

In the present study, we hypothesize that *UCA1* knockdown inhibits DDP resistance and malignant progression in cervical cancer cells via the miR-195-5p/IKBKB axis and that UCA1 might regulate NF-қB, BNIP3, and BNIP3∆ex2 expressions in these cells.

## Materials and Methods

### Clinical specimens

Tissue samples were collected from cervical cancer patients who received DDP chemotherapy between March 2020 and October 2020 at the Affiliated Hospital of Guizhou Medical University. All cervical cancer patients underwent radical resection and received DDP chemotherapy for at least 3 courses, usually 6–8 courses, after surgery. The follow-up period ranged from 10–60 months post-surgery, with a median follow-up time of 24 months. The tissue samples were divided into DDP-resistant and DDP-sensitive groups on the basis of patient DDP chemotherapy prognosis. All patients provided signed informed consent, and the study was reviewed and approved by the hospital’s Medical Ethics Committee.

### Cell culture

Human HeLa cells were purchased from IZSLER (Brescia, Italy), and incubated in DMEM (Gibco, Carlsbad, USA) containing 10% fetal bovine serum (FBS; Sigma-Aldrich, St Louis, USA), 1 mM sodium pyruvate (Carlo Erba, Milan, Italy), 0.04 mg/mL gentamicin (Gibco), and 2 mM L-glutamine (Gibco) at 37°C with 5% CO _2_.

### Construction of DDP-resistant HeLa cell lines

DDP-resistant HeLa cells were established using a concentration gradient increment method (DDP from 0.1 μg/mL to 2.0 μg/mL). Briefly, HeLa cells were inoculated in a culture dish, and the growth rate was approximately 70%. The cells were incubated with culture medium containing 0.1 μg/mL DDP for 24 h. The drug-containing culture medium was discarded, and fresh culture medium was added to continue the culture. Once the cells returned to normal growth, they were digested and passaged. The cells were then treated with DDP (0.2 μg/mL) for 24 h. This process was repeated, and the DDP concentration was gradually increased until the cells could no longer tolerate it. Continuous induction of cells resulted in a stable DDP-resistant HeLa cell line named HeLa/DDP. The cells were cultured in 2.0 μg/mL DDP-containing culture medium for 24 h to maintain resistance. The cells were cultured in DDP-free culture medium for at least 3 days before the experiment.

### Cell transfection

Plasmids overexpressing UCA1 (OE-lncRNA UCA1), BNIP3Δex2 (OE-BNIP3Δex2), BNIP3 (OE-BNIP3), or IKBKB (OE-IKBKB) and an empty vector were purchased from Biosyntech (Suzhou, China). UCA1 shRNAs (sh-lncRNA UCA1), BNIP3Δex2 shRNAs (sh-BNIP3Δex2), BNIP3 shRNAs (sh-BNIP3), IKBKB shRNAs (sh-IKBKB), miR-195-5p mimics, and the miR-195-5p inhibitor were obtained from GenePharma (Shanghai, China). HeLa/DDP cells were transfected with shRNAs, overexpression plasmids, miR-195-5p mimics (50 nM), or the miR-195-5p inhibitor (50 nM) using Lipofectamine 3000 (Invitrogen, Carlsbad, USA) following the kit instructions. The shRNA and miRNA sequences are shown in Table 1.

**Table:** **Table 1** The sequences of shRNA and miRNA used in this study

Name	Sequence (5′→3′)
sh-lncRNA UCA1	CCGCCTATAAAAAGGATTATATC
sh-BNIP3△ex2	CACUGUGACAGUCUGAGGA
sh-BNIP3	UCGCAGACACCACAAGAUA
sh-NC	AAGAAACCATG CAAAGTAAGGTT
sh-IKBKB	CAGAGGTTAATACACAAAATTAT
miR-195-5p mimics	UAGCAGCACAGAAAUAUUGGC
miR-195-5p inhibitor	CCAATATTTCTGTGCTGCT
NC mimics	UUCUCCGAACGUGUCACGUTT
NC inhibitor	CAGUACUUUUGUGUAGUACAA

### qRT-PCR analysis

Total RNA was extracted from the transfected HeLa/DDP cells or tumor tissues using Trizol (Invitrogen). The levels of the lncRNAs BNIP3 and BNIP3Δex2 were examined via a reverse transcription kit (Servicebio, Shanghai, China) with 2× Realstar Fast SYBR qPCR Mix (GenStar, Beijing, China). The levels of miR-195-5p were monitored using a TaqMan MicroRNA Reverse Transcription kit (Applied Biosystems, Foster City, USA) and Taqman Universal Master Mix II (Applied Biosystems). Gene expression levels were quantified via the 2 ^–ΔΔCt^ method. The primer sequences are presented in Table 2.

**Table:** **Table 2** The sequences of primers for qRT-PCR

Gene	Forward primer (5′→3′)	Reverse primer (5′→3′)
*lncRNA UCA1*	CTCTCCTATCTCCCTTCACTGA	CTCTCCTATCTCCCTTCACTGA
*BNIP3△ex2*	GCCGGAATTCATGTCGCAGAGCGGGGAG	CGGCGCTCGAGTCAGGATACTTTCAACTTCTCTTCTTCTCTC
*BNIP3*	GCCGGAATTCATGTCGCAGAGCGGGGAG	CGGCGCTCGAGTCAGAAGGTGCTAGTGGAAGTTGTC
*ABAB1*	TGACAGCTACAGCACGGAAG	TGGCAATGCGTTGTTTCTGG
*GAPDH*	CACCATTGGCAATGAGCGGTTC	AGGTCTTTGCGGATGTCCACGT
*miR-195-5p*	GCGTAGCAGCACAGAAATATTGGC	CTGTCGTCGTAGAGCCAGGGAA
*U6*	CTCGCTTCGGCAGCACATA	AACGATTCACGAATTTGCGT

### Western blot analysis

Total protein was collected from transfected HeLa/DDP cells or tumor tissues using RIPA buffer (Beyotime, Shanghai, China). After quantification with a BCA kit (ECOTOP, Guangzhou, China), proteins (40 μg) were separated through electrophoresis and transferred to a PVDF membrane (Millipore, Billerica, USA). Membranes were then blocked for 2 h, incubated with primary antibodies overnight at 4°C, and then incubated with HRP-conjugated Affinipure goat anti mouse (SA-00001-1) or HRP-conjugated Affinipure goat anti rabbit secondary antibodies (1:2000, SA-00001-2; Proteintech, Wuhan, China) for 2 h. Target proteins were visualized after treatment with enhanced chemiluminescence (ECL) reagent (Millipore). The primary antibodies used included anti-BNIP3 (1:1000, #44060; Cell Signaling, Danvers, USA), anti-p-gp (1:1000, #22336-1-AP; Proteintech), anti-IKBKB (1:500, #0024903; Chuangwei Biotechnology, Guangzhou, China), anti-p65 (1:1000, #23004899; Chuangwei Biotechnology), anti-p-p65 (1:1000, #00119680; Chuangwei Biotechnology), anti-Caspase3 (1:1000, #66470-2-Ig; Proteintech), anti-PARP1 (1:1000; Abcam, #ab191217), anti-C-PARP1 (1:1000, #ab110315; Abcam, Cambridge, UK), anti-β-actin (1:1000, #GB15001; Servicebio), and anti-histone (1:1000, # BF9211; Affinity, Cincinnati, USA) antibodies.

### CCK-8 assay

HeLa/DDP cells were washed and collected, and their density was adjusted to 5 × 10 ^4^ cells/mL. The cells (100 μL) from each group were seeded in 96-well plates and incubated for 24 h. Then, 10 μL of CCK-8 (Beyotime) was added to the cells. After 2 h, the OD was measured using a microplate reader (ZDM-1096A; Droide, Shanghai, China) at 450 nm. The IC _50_ values (μg/mL) of the drugs in HeLa and HeLa/DDP cells were calculated.

### Wound healing assay

HeLa/DDP cells from each group were seeded in 6-well plates and cultured until they reached approximately 80%–90% confluence. Once a monolayer formed, a sterile 200-μL pipette tip was used to create a linear scratch perpendicular to the cell layer. The cells were gently washed twice with PBS to remove detached cells and debris, and serum-free medium was added to the wells to minimize the effect of cell proliferation. The cells were then incubated at 37°C with 5% CO _2_ for 24 h to allow for migration into the wound area. Images of the scratched area were captured at 0 or 24 h using a light microscope (Olympus, Tokyo, Japan).

### Transwell assay

A 24-well chamber (8-μm pores; Corning, New York, USA) was pre-coated with Matrigel for 30 min at 37°C. The upper chamber was filled with transfected HeLa/DDP cells (300 μL, 1 × 10 ^5^ cells/mL) in plain medium, while the bottom chamber was filled with 600 μL of medium containing 10% FBS. After 24 h of incubation at 37°C, the invading cells were fixed with 4% paraformaldehyde, stained with 0.5% crystal violet, and counted under a light microscope (Olympus).

### Flow cytometry

The cells were collected and washed. Then, the cells (1 × 10 ^5^ cells/well) were stained with 5 μL of Annexin V-FITC and 5 μL of PI (KeyGen, Shanghai, China) in the dark for 30 min. The apoptosis rate was determined using a flow cytometer (FACSCanto II; BD Biosciences, Franklin Lakes, USA).

### RNA immunoprecipitation (RIP) assay

The interaction between NK-κB p65 and *BNIP3* mRNA was confirmed using an RIP kit (Thermo Fisher Scientific, Waltham, USA). Briefly, the cells were harvested in RIP lysis buffer. Lysed samples were mixed with magnetic beads ligated with anti-NK-κB p65 or control IgG. After precipitation, BNIP3 levels were analyzed by qRT-PCR.

### Dual-luciferase reporter gene assay

Binding fragments of UCA1 and miR-195-5p or miR-195-5p and IKBKB were predicted using the TargetScan database ( http://www.targetscan.org/). The UCA1 and IKBKB fragments were amplified via PCR and inserted into the psiCHECK reporter vector to construct the wild-type (WT) UCA1 and WT-IKBKB plasmids. A PCR site-directed mutagenesis assay was applied to create mutant (Mut) plasmids of UCA1 (CUGCC mutated to AUGAA) and IKBKB (GCUGC mutated to CAUCA). HeLa/DDP cells were transfected with WT or Mut plasmids and with the miR-195-5p inhibitor or mimics via Lipofectamine ^TM^ 3000 (Invitrogen) for 48 h. Luciferase activity was measured via a dual-luciferase reporter system (Promega, Madison, USA).

### Experimental animals

Female BALB/c mice (6–8 weeks old) were purchased from the Lanzhou Veterinary Research institute Chinese Academy of Agricultural Sciences (Lanzhou, China) and maintained under SPF conditions. Stably transfected HeLa/DDP cells (2 × 10 ^6^ cells) were injected subcutaneously into the lateral skin of nude mice ( *n* = 6). After four weeks, the mice were euthanized by cervical dislocation. The tumors were removed, photographed, and weighed, and the tumor volumes were measured. The animal experiments were approved by the Animal Care and Use Committee of Guizhou Medical University.

### H&E staining

The tissue samples were fixed in 10% neutral formalin, embedded in paraffin, and sectioned serially at a thickness of 4 μm. The sections were deparaffinized with xylene for 5 min (repeated twice) and then treated with 100%, 95%, 80%, 70%, or distilled water for 3 min each. The sections were treated with hematoxylin (1 min), differentiation solution (30 s), tap water (15 min), eosin staining (1 min), and graded alcohol (70%, 80%, 90%, and 100%) for rapid dehydration and xylene (1 min). After sealing with neutral gum, histopathological structures were observed under a light microscope (Olympus).

### TUNEL staining

The sections were deparaffinized and rehydrated, followed by microwave repair in citric acid for 8 min. The sections were then treated with fluorescent TUNEL solution (reagent A:reagent B = 1:30; Yeasen, Shanghai, China) for 1 h at 37°C. After wash with PBS, DAPI was added to the sections and incubated for 15 min. The sections were sealed with glycerin gelatin and photographed under a fluorescence microscope (Nikon Eclipse C1; Nikon, Tokyo, Japan). The nuclei appeared blue, and apoptotic cells appeared green.

### Immunohistochemistry (IHC)

The sections were deparaffinized with xylene and rehydrated with gradient ethanol. High-pressure antigenic repair was performed using citrate. After washing, the sections were treated with 3% H _2_O _2_, blocked with goat serum (Gibco) for 1 h, and incubated with anti-PCNA (1:20; Abcam) or anti-Ki67 (1:20; Abcam) antibodies overnight at 4°C. After washing, the sections were treated with a biotin-labelled secondary antibody (1:400; Abcam) for 1 h, followed by incubation with HRP-conjugated streptavidin, and then stained with 3,3′-diaminobenzidine (DAB; Maxim Biotech, Fuzhou, China) and hematoxylin (Sigma-Aldrich) for 2 min, and differentiated with hydrochloric acid ethanol. Following dehydration and transparency, the protein expression intensity was observed under a light microscope (Olympus).

### Statistical analysis

Data are expressed as the mean ± SD from three independent experiments. Statistical analyses were performed using SPSS 19.0 (SPSS; IBM, Chicago, USA) with a Student’s *t* test (for two groups) or one-way ANOVA (for multiple groups). *P*  < 0.05 was considered to indicate statistical significance.

## Results

### UCA1 and BNIP3Δex2 are highly expressed, and BNIP3 is expressed at low levels in DDP-resistant cervical cancer

**Figure 1:**
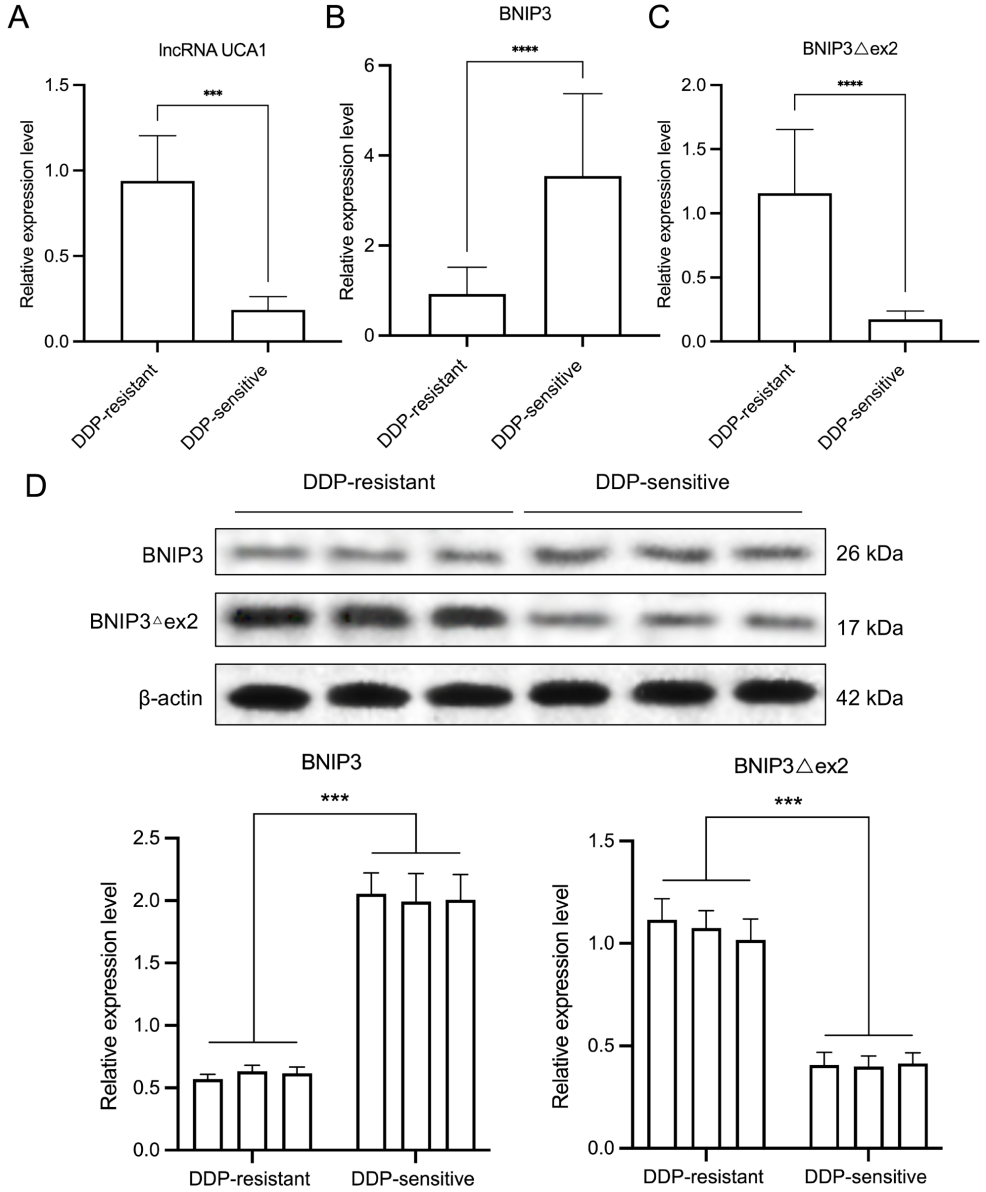
Figure 1 The expressions of UCA1, BNIP3Δex2, and BNIP3 in DDP-resistant and sensitive cervical cancer tissues (A–C) qRT-PCR was used to assess the changes in the expressions of UCA1 (A), BNIP3 (B), and BNIP3Δex2 (C) in cervical cancer tissues from DDP-resistant (n = 30) and DDP-sensitive patients (n = 30). (D) BNIP3 and BNIP3△ex2 protein expressions in cervical cancer tissues from DDP-resistant (n = 3) and DDP-sensitive patients (n = 3) was monitored via western blot analysis and quantified. ***P < 0.001, ****P < 0.0001.

To verify the expressions of UCA1, BNIP3Δex2, and BNIP3 in DDP-resistant cervical cancer, tumor tissues from patients were divided into DDP-resistant or DDP-sensitive groups on the basis of DDP chemotherapy prognosis. qRT-PCR results indicated that the levels of *UCA1* and *BNIP3Δex2* were higher in the DDP-resistant group than in the DDP-sensitive group, whereas the *BNIP3* level was lower in the DDP-resistant group ( Figure 1A–C). Moreover, compared with that in the DDP-sensitive group, BNIP3 was expressed at low levels, and BNIP3Δex2 was highly expressed in the DDP-resistant group ( Figure 1D). These data suggest that UCA1, BNIP3Δex2, and BNIP3 are involved in the DDP resistance of cervical cancer.

### UCA1 and BNIP3Δex2 are upregulated, whereas BNIP3 is downregulated in DDP-resistant HeLa cells

To further validate the possible role and mechanism of UCA1 in DDP-resistant cervical cancer, a DDP-resistant cell model was established by culturing HeLa cells with increasing concentrations of DDP. The CCK-8 results suggested that the IC _50_ of DDP in HeLa cells was 4.280 μg/mL ( Supplementary Figure S1A); the IC _50_ of DDP in HeLa/DPP cells was 54.05 μg/mL after 48 h of incubation ( Supplementary Figure S1B). The qRT-PCR results indicated that the mRNA level of a drug resistance-related gene ( *p-gp*) in the HeLa/DPP cells was higher than that in the HeLa cells ( Supplementary Figure S1C). The protein level of p-gp was also higher in HeLa/DPP cells than in HeLa cells ( Supplementary Figure S1D). These data confirmed the successful generation of DDP-resistant HeLa cells.

**Figure 2:**
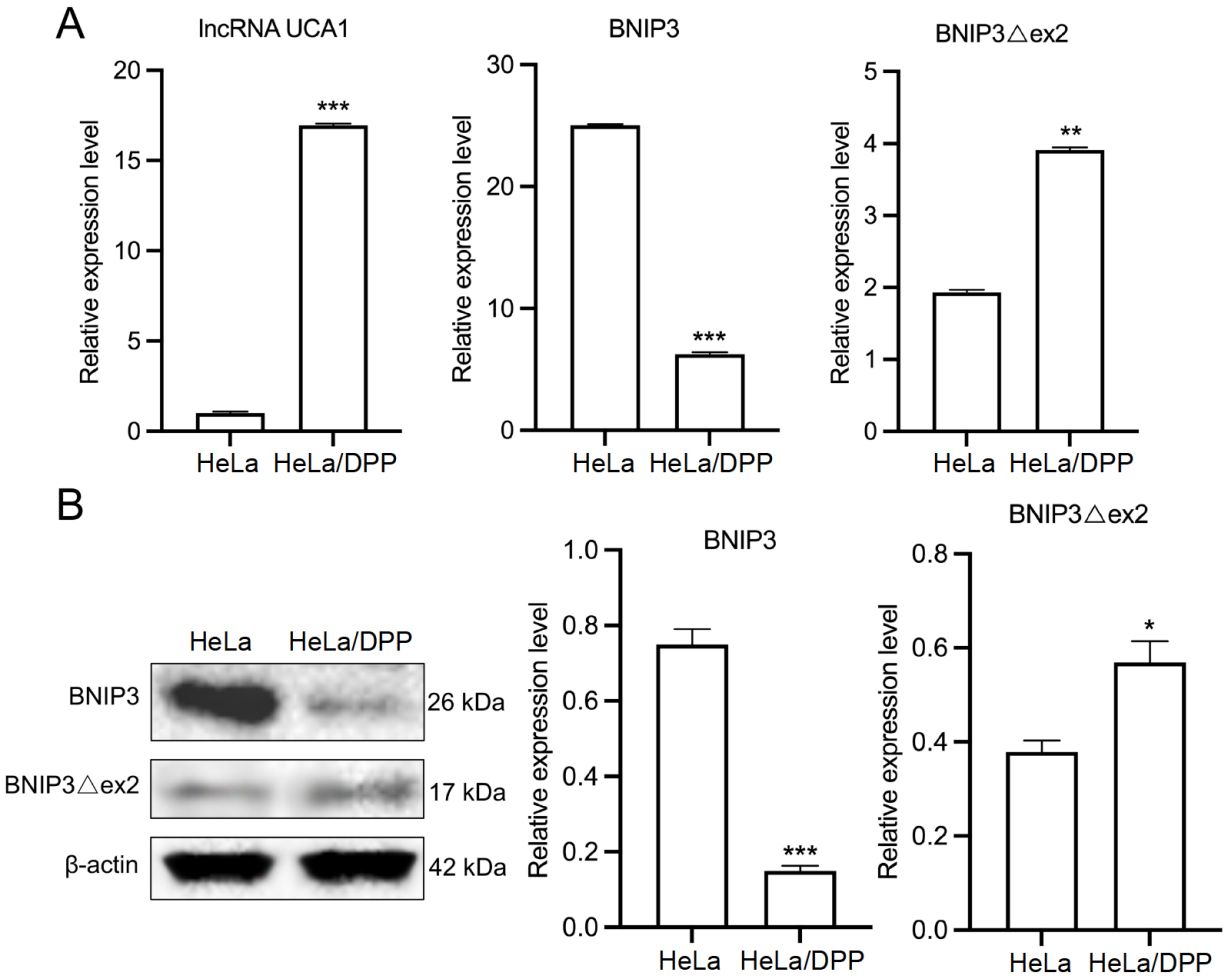
Figure 2 Expressions of UCA1, BNIP3, and BNIP3Δex2 in HeLa/DDP cells (A) qRT-PCR analysis of the levels of UCA1, BNIP3, and BNIP3Δex2 in HeLa and HeLa/DDP cells. (B) Western blot analysis of BNIP3 and BNIP3Δex2 protein expressions in HeLa and HeLa/DDP cells. *P < 0.05, **P < 0.01, ***P < 0.001.

Next, the expressions of *UCA1*, *BNIP3Δex2*, and *BNIP3* were verified in HeLa/DDP cells. Compared with those in HeLa cells, the mRNA levels of *UCA1* and *BNIP3Δex2* were significantly increased, whereas the mRNA level of *BNIP3* was markedly decreased in HeLa/DPP cells ( Figure 2A). Similarly, the BNIP3 protein was downregulated, and the BNIP3Δex2 protein was upregulated in HeLa/DPP cells ( Figure 2B).

### *UCA1* is a cisplatin resistance-related gene in cervical cancer

**Figure 3:**
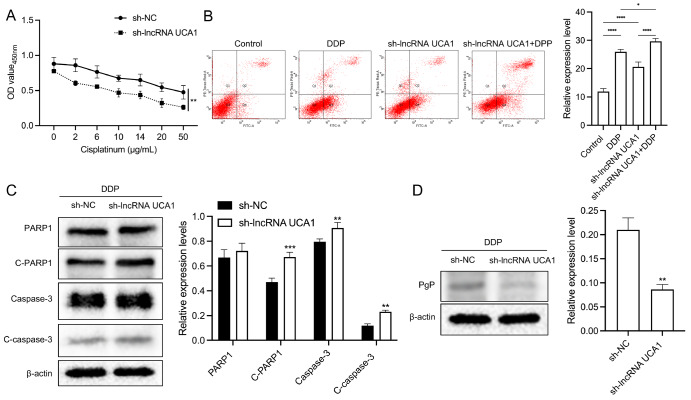
Figure 3 *UCA1* silencing suppresses cervical cancer cell resistance to cisplatin (A) CCK-8 assay of the viability of UCA1-silenced HeLa cells treated with the indicated DDP concentrations for 24 h. (B) Cell apoptosis was examined using a flow cytometry in UCA1-silenced HeLa cells treated with DDP. (C) Western blot analysis of PARP1, cleaved-PARP1, caspase 3, and cleaved-caspase 3 levels in UCA1-silenced HeLa cells after DDP treatment. (D) p-gp expression was determined by western blot analysis in UCA1-silenced HeLa cells treated with DDP. **P < 0.01, ***P < 0.001.

HeLa cells were exposed to 0, 2, 6, 10, 14, 20, or 50 μg/mL DDP for 24 h. CCK-8 assay results demonstrated that *UCA1* silencing restored HeLa cell sensitivity to DDP ( Figure 3A). The data also indicated that DDP treatment or *UCA1* silencing markedly increased the number of apoptotic cells; after DDP treatment, HeLa cells transfected with UCA1 shRNAs also exhibited increased apoptosis ( Figure 3B). The data indicated that UCA1 overexpression promoted cell proliferation ( Supplementary Figure S2A) and suppressed apoptosis in HeLa/DDP cells ( Supplementary Figure S2B). Additionally, western blot analysis data revealed that the levels of cleaved PARP1, caspase 3, and cleaved caspase 3 were higher in *UCA1*-silenced HeLa cells than in sh-NC-transfected cells after DDP treatment ( Figure 3C). *UCA1* silencing also reduced p-gp expression in HeLa/DDP cells ( Figure 3D).

### Effects of UCA1, BNIP3Δex2, and BNIP3 on the malignant processes of HeLa/DPP cells

**Figure 4:**
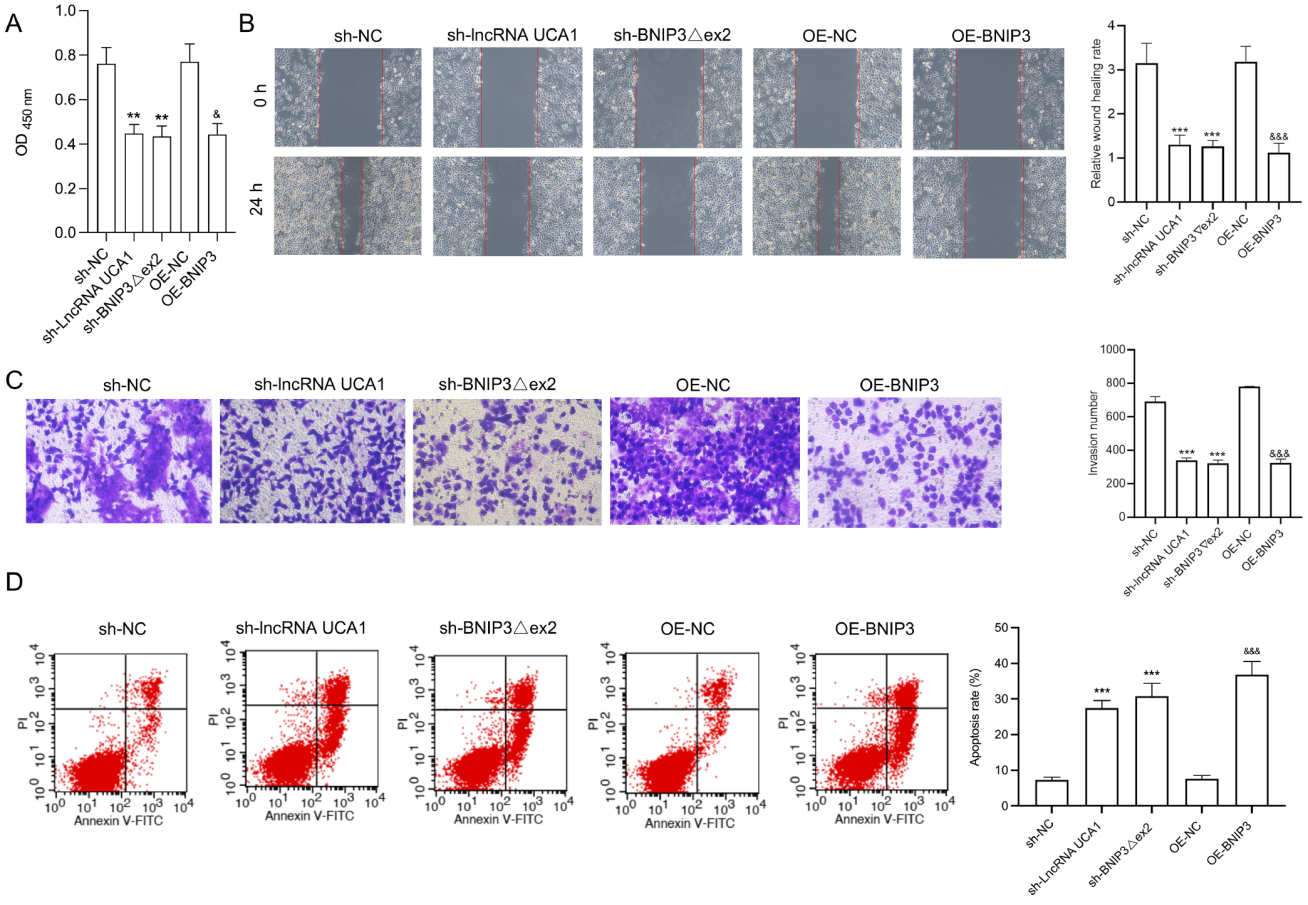
Figure 4 *UCA1* silencing, *BNIP3Δex2* silencing, and *BNIP3* overexpression suppress proliferation, migration, and invasion but accelerate apoptosis in HeLa/DDP cells HeLa/DPP cells were transfected with UCA1 shRNAs, BNIP3Δex2 shRNAs, or BNIP3 overexpression plasmids. (A) CCK-8 assay of cell proliferation. (B) Wound healing assay of cell migration. (C) Transwell assay of cell invasion. (D) Flow cytometry assay of cell apoptosis. **P < 0.01, ***P < 0.001 vs the sh-NC group; &P < 0.05, &&&P < 0.001 vs the OE-NC group.

**Figure 5:**
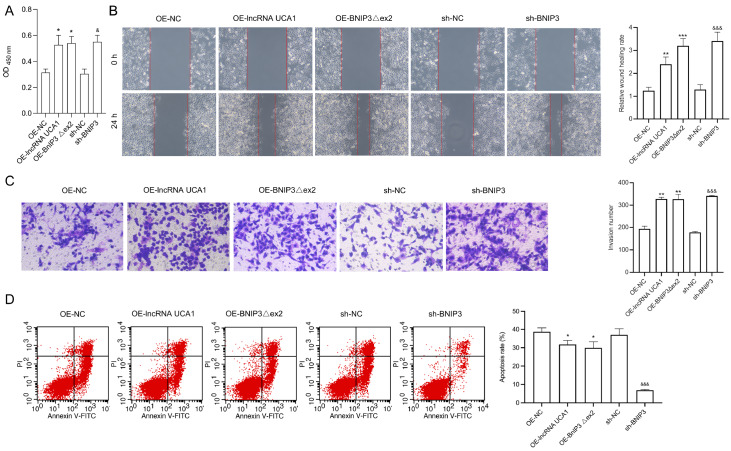
Figure 5 UCA1 overexpression, BNIP3Δex2 overexpression, and *BNIP3* silencing enhance proliferation, migration, and invasion but suppress apoptosis in HeLa/DDP cells HeLa/DPP cells were transfected with the UCA1 overexpression plasmid, BNIP3Δex2 overexpression plasmid, or BNIP3 shRNAs. (A) CCK-8 analysis of cell proliferation. (B) Wound healing assay of cell migration. (C) Transwell assay of cell invasion. (D) Flow cytometry assay of cell apoptosis. *P < 0.05, **P < 0.01, ***P < 0.001 vs the OE-NC group; &P < 0.05, &&&P < 0.001 vs the sh-NC group.

To investigate the effects of UCA1, BNIP3Δex2, and BNIP3 on the malignant behaviors of HeLa/DDP cells, overexpression plasmids and shRNAs for these genes were transfected into HeLa/DPP cells. The qRT-PCR data revealed that *UCA1* expression was notably increased in HeLa/DPP cells after transfection with the UCA1 overexpression plasmid and notably decreased after transfection with UCA1 shRNA, indicating successful overexpression or silencing of *UCA1* in HeLa/DPP cells ( Supplementary Figure S3A). Similarly, *BNIP3Δex2* and *BNIP3* expressions were also successfully overexpressed or silenced in HeLa/DPP cells by transfection ( Supplementary Figure S3B,C). HeLa/DPP cells were then transfected with sh-lncRNA UCA1, sh-BNIP3Δex2, or BNIP3 overexpression plasmids. The CCK-8 results revealed that *UCA1* silencing, *BNIP3Δex2* silencing, and *BNIP3* overexpression significantly reduced the proliferation of HeLa/DPP cells ( Figure 4A). *UCA1* silencing, *BNIP3Δex2* silencing, and *BNIP3* overexpression also prevented the migration and invasion of HeLa/DPP cells ( Figure 4B,C). Additionally, the percentage of apoptotic HeLa/DPP cells was markedly increased in the *UCA1*-silenced group, *BNIP3Δex2*-silenced group, and BNIP3-overexpressing group ( Figure 4D). Conversely, UCA1 overexpression, BNIP3Δex2 overexpression, and *BNIP3* silencing accelerated proliferation, migration, and invasion while suppressing the apoptosis of HeLa/DPP cells ( Figure 5).

### UCA1 targets miR-195-5p, miR-195-5p targets IKBKB, and UCA1 overexpression upregulates BNIP3Δex2 and p-p65 but downregulates BNIP3 in HeLa/DDP cells

**Figure 6:**
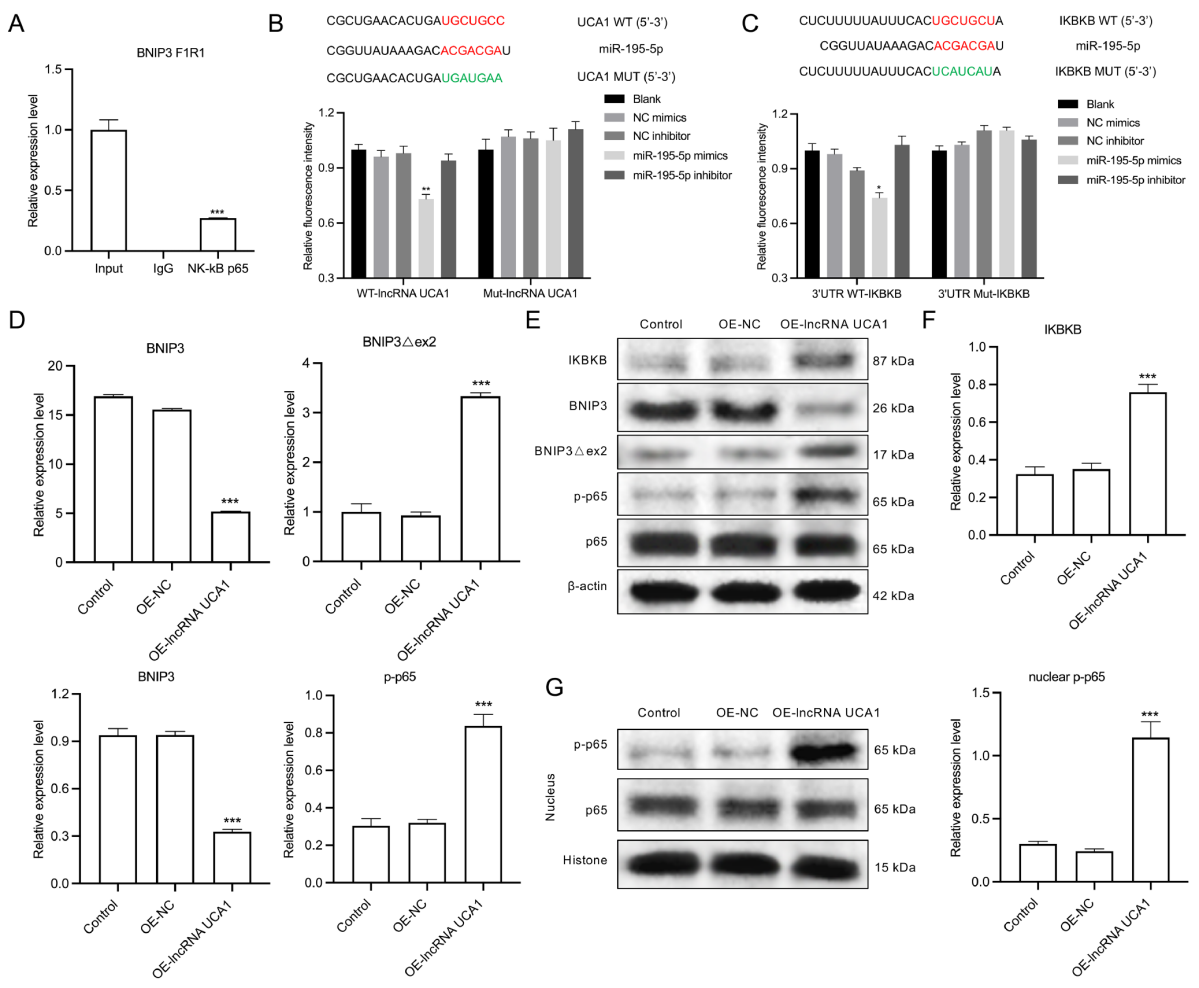
Figure 6 The regulatory effects of UCA1 on miR-195-5p, IKBKB, BNIP3Δex2, BNIP3, and p-p65 in HeLa/DDP cells (A) Interaction of NK-κB p65 and BNIP3 mRNA was confirmed via a RIP assay in HeLa/DDP cells. (B) Dual-luciferase reporter gene assay was used to monitor the interaction between UCA1 and miR-195-5p. (C) Dual-luciferase reporter gene assay to confirm the interaction between miR-195-5p and IKBKB mRNA. (D) qRT-PCR assay of BNIP3 and BNIP3Δex2 expressions in UCA1-overexpressing HeLa/DDP cells. (E) Western blot analysis of IKBKB, BNIP3, BNIP3Δex2, p65, and p-p65 expressions in UCA1-overexpressing HeLa/DDP cells. (F) Quantification of protein bands. (G) Western blot analysis of p65 and p-p65 expressions in the nucleus of UCA1-overexpressing HeLa/DDP cells. **P < 0.01, ***P < 0.001.

We subsequently analyzed and verified the possible regulatory mechanism of UCA1 in HeLa/DPP cells. First, we analyzed the regulatory roles of UCA1, miR-195-5p, IKBKB, and NF-κB in HeLa/DPP cells. The RIP results revealed that BNIP3 was detected in the NF-κB p65 antibody group but not in the IgG control group, indicating that BNIP3 can bind to NF-κB ( Figure 6A). After database prediction and literature search, we speculated that miR-195-5p could target and bind to UCA1 and IKBKB. Then, mutant IKBKB and UCA1 were designed and verified by a luciferase reporter assay. The dual-luciferase reporter gene results indicated that overexpression of miR-195-5p decreased the luciferase activity of WT UCA1 but not that of Mut-UCA1 ( Figure 6B); overexpression of miR-195-5p also inhibited the luciferase activity of WT-IKBKB but did not change the luciferase activity of Mut-IKBKB ( Figure 6C). These data suggested a direct interaction between UCA1 and miR-195-5p and between miR-195-5p and IKBKB. Additionally, UCA1 overexpression significantly decreased BNIP3 level and increased BNIP3Δex2 level in HeLa/DPP cells ( Figure 6D). These data further suggested that BNIP3 and BNIP3Δex2 are downstream regulatory proteins of UCA1. Western blot analysis revealed that UCA1 overexpression dramatically elevated the protein levels of IKBKB, BNIP3Δex2, and p-p65 while reducing BNIP3 level in HeLa/DPP cells ( Figure 6E,F). Moreover, UCA1 overexpression prominently increased p-p65 level in the nucleus of HeLa/DPP cells ( Figure 6G).

### miR-195-5p overexpression downregulates IKBKB, BNIP3Δex2, and p-p65 expressions but upregulates BNIP3 expression in HeLa/DDP cells

**Figure 7:**
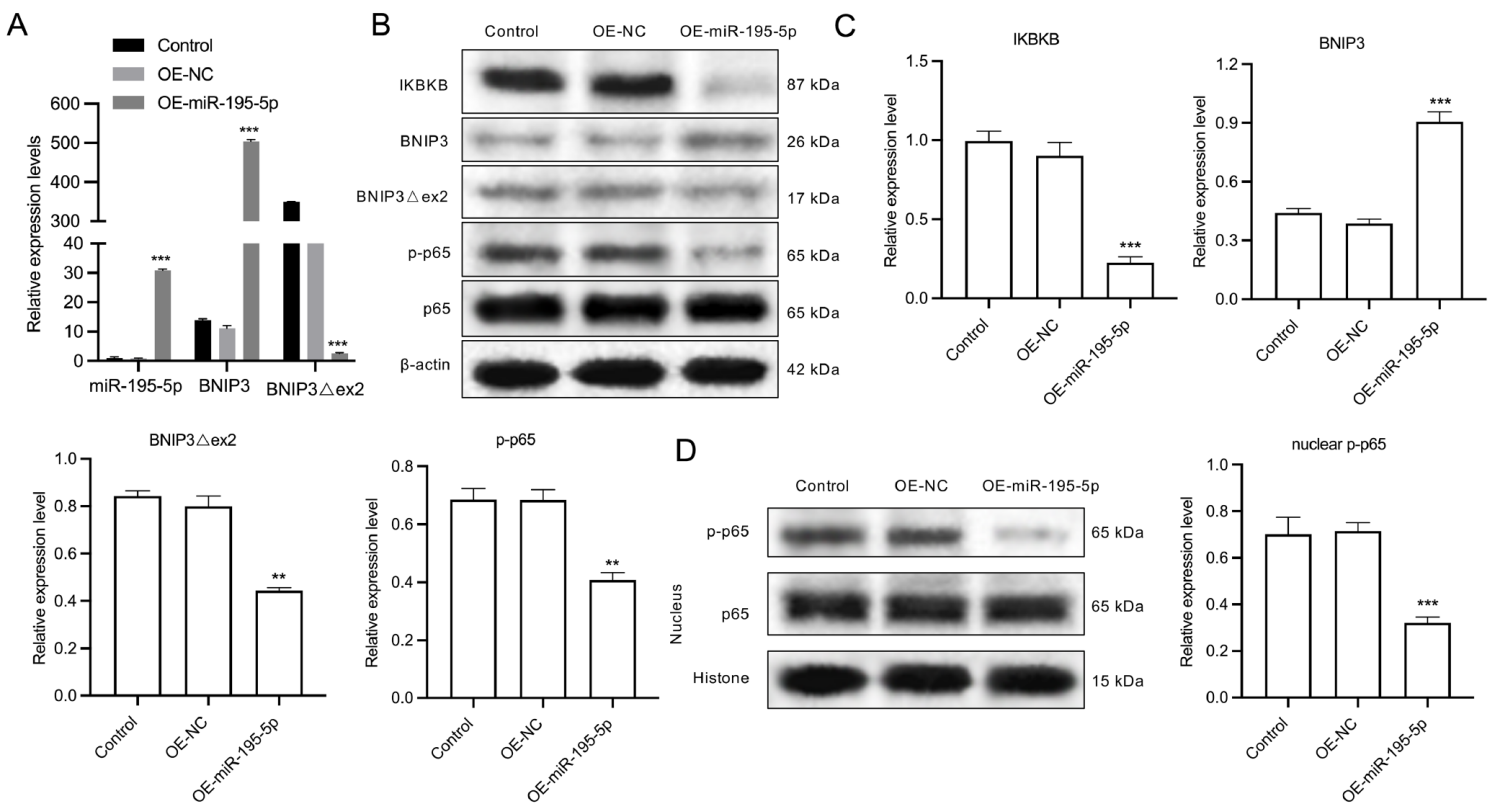
Figure 7 The regulatory effects of miR-195-5p on IKBKB, BNIP3Δex2, BNIP3, and p-p65 in HeLa/DDP cells (A) qRT-PCR analysis of miR-195-5p, BNIP3, and BNIP3Δex2 expressions in miR-195-5p-overexpressing HeLa/DDP cells. (B) Western blot analysis of IKBKB, BNIP3, BNIP3Δex2, p65, and p-p65 expressions in miR-195-5p-overexpressing HeLa/DDP cells. (C) Quantification of protein bands. (D) Western blot analysis of p65 and p-p65 expressions in the nucleus of miR-195-5p-overexpressing HeLa/DDP cells. **P < 0.01, ***P < 0.001.

We also verified the regulatory effect of miR-195-5p on downstream proteins in HeLa/DDP cells. The results revealed that miR-195-5p overexpression significantly upregulated miR-195-5p and BNIP3 but downregulated BNIP3Δex2 at the mRNA level in HeLa/DDP cells ( Figure 7A). Moreover, miR-195-5p overexpression markedly decreased IKBKB, BNIP3Δex2, and p-p65 protein levels but increased BNIP3 protein level in HeLa/DDP cells ( Figure 7B,C). In addition, miR-195-5p overexpression reduced p-p65 protein level in the nuclei of the HeLa/DDP cells ( Figure 7D).

### IKBKB overexpression upregulates BNIP3Δex2 and p-p65 expressions but downregulates BNIP3 expression in HeLa/DDP cells

**Figure 8:**
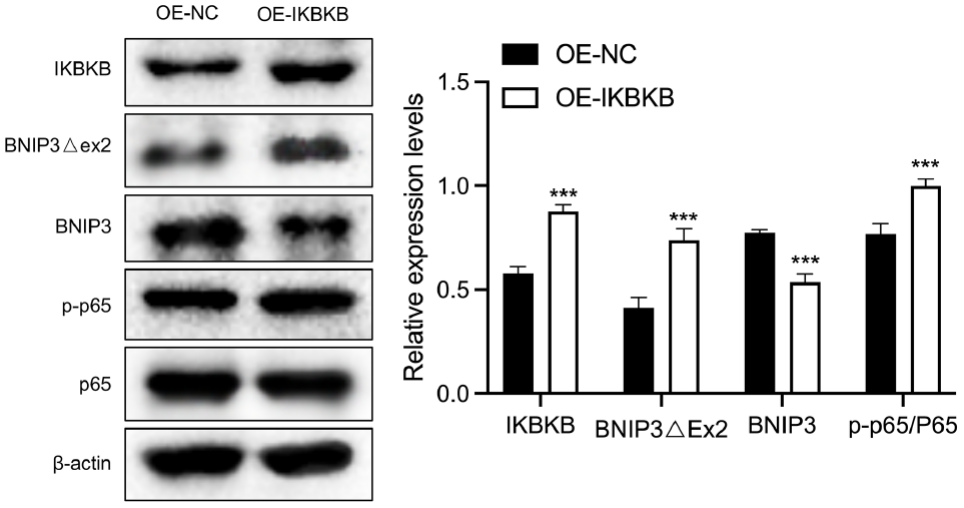
Figure 8 The impact of IKBKB overexpression on BNIP3Δex2, BNIP3, and p-p65 in HeLa/DDP cells Western blot analysis of IKBKB, BNIP3Δex2, BNIP3, p65, and p-p65 expressions in IKBKB-overexpressing HeLa/DDP cells. ***P < 0.001.

To further elucidate the role of IKBKB in regulating BNIP3 and its isoforms, we examined the effects of IKBKB overexpression on the expressions of BNIP3 and related proteins in HeLa/DDP cells. Western blot analysis data revealed that, compared with those in the OE-NC group, BNIP3Δex2 and p-p65 expressions were increased, whereas BNIP3 expression was decreased in the HeLa/DDP cells ( Figure 8).

### *UCA1* knockdown inhibits the malignancy of HeLa/DPP cells by targeting the miR-195-5p/IKBKB axis

**Figure 9:**
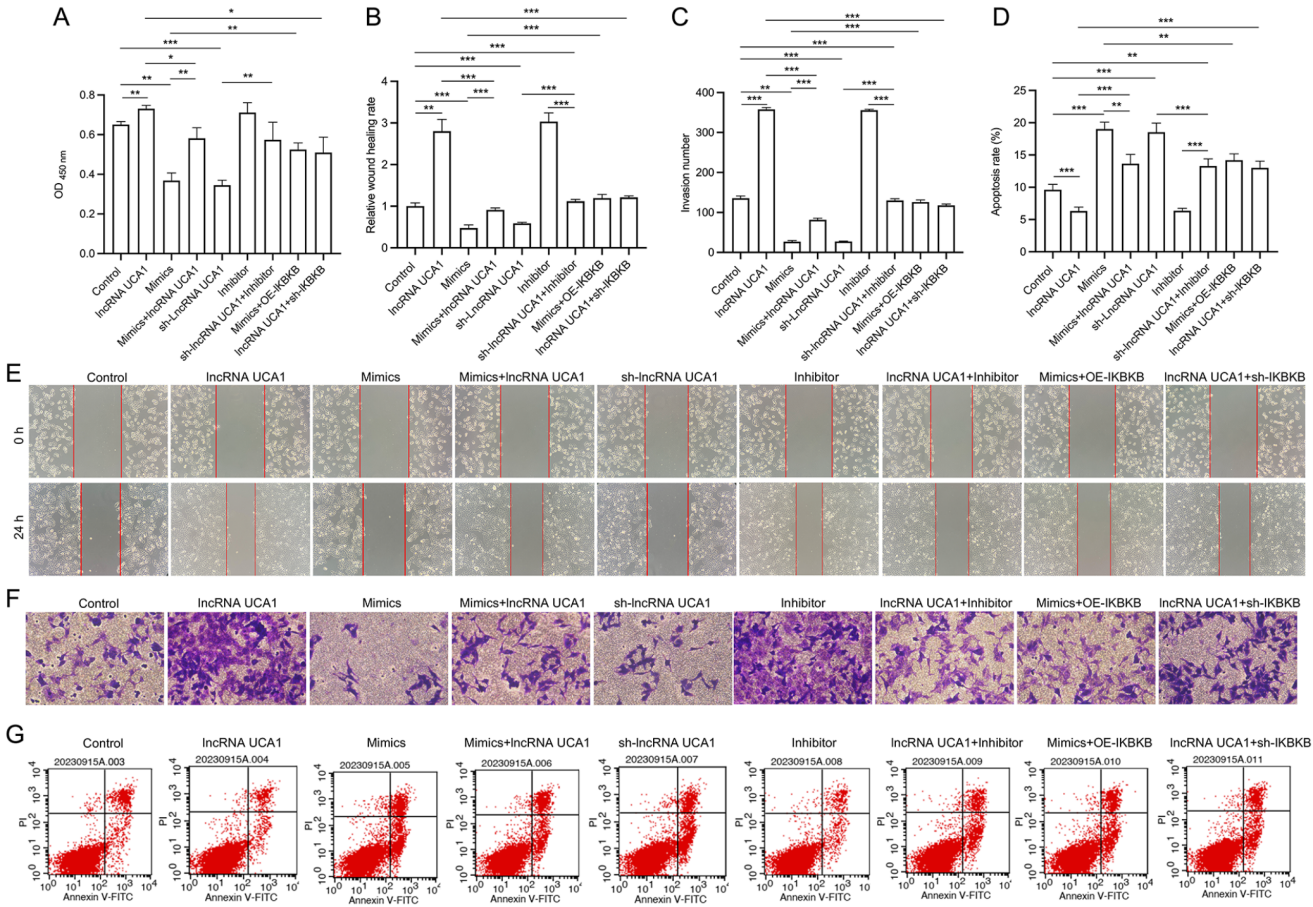
Figure 9 Aberrant expression of UCA1 affects the malignant process of HeLa/DDP cells by altering miR-195-5p and IKBKB (A) CCK-8 assay of cell proliferation in the transfected HeLa/DPP cells. (B) Wound healing rate of the transfected HeLa/DPP cells. (C) Quantitative analysis of the number of invasive cells. (D) Quantitative analysis of the percentage of apoptotic cells. (E) Wound healing assay of the migration of the transfected HeLa/DPP cells. (F) Transwell assay of cell invasion. (G) Flow cytometry analysis of cell apoptosis. *P < 0.05, **P < 0.01, ***P < 0.001.

Next, we conducted rescue experiments to analyze the role of the UCA1/miR-195-5p/IKBKB axis in HeLa/DPP cell progression. The CCK-8 data revealed that overexpression of UCA1 or inhibition of miR-195-5p promoted cell proliferation, whereas overexpression of miR-195-5p or silencing of *UCA1* attenuated cell proliferation. miR-195-5p overexpression reversed the cell proliferation induced by UCA1 overexpression; miR-195-5p inhibition reversed the inhibitory effect of *UCA1* silencing on cell proliferation; IKBKB overexpression attenuated the inhibitory effect of miR-195-5p on cell proliferation; and *IKBKB* silencing reversed the stimulatory effect of UCA1 overexpression on cell proliferation in HeLa/DPP cells ( Figure 9A). Additionally, *UCA1* overexpression accelerated the migration and invasion of HeLa/DPP cells by downregulating miR-195-5p level or upregulating IKBKB level; *UCA1* silencing inhibited migration and invasion by increasing miR-195-5p expression; and miR-195-5p overexpression attenuated migration and invasion by downregulating IKBKB ( Figure 9B,C,E,F). Furthermore, *UCA1* overexpression prevented HeLa/DPP cell apoptosis by reducing miR-195-5p expression or increasing IKBKB expression; *UCA1* silencing accelerated HeLa/DPP cell apoptosis by increasing miR-195-5p expression; and miR-195-5p overexpression enhanced HeLa/DPP cell apoptosis by downregulating IKBKB level ( Figure 9D,G).

### UCA1 overexpression plays a carcinogenic role in a nude mouse tumor model by targeting miR-195-5p

**Figure 10:**
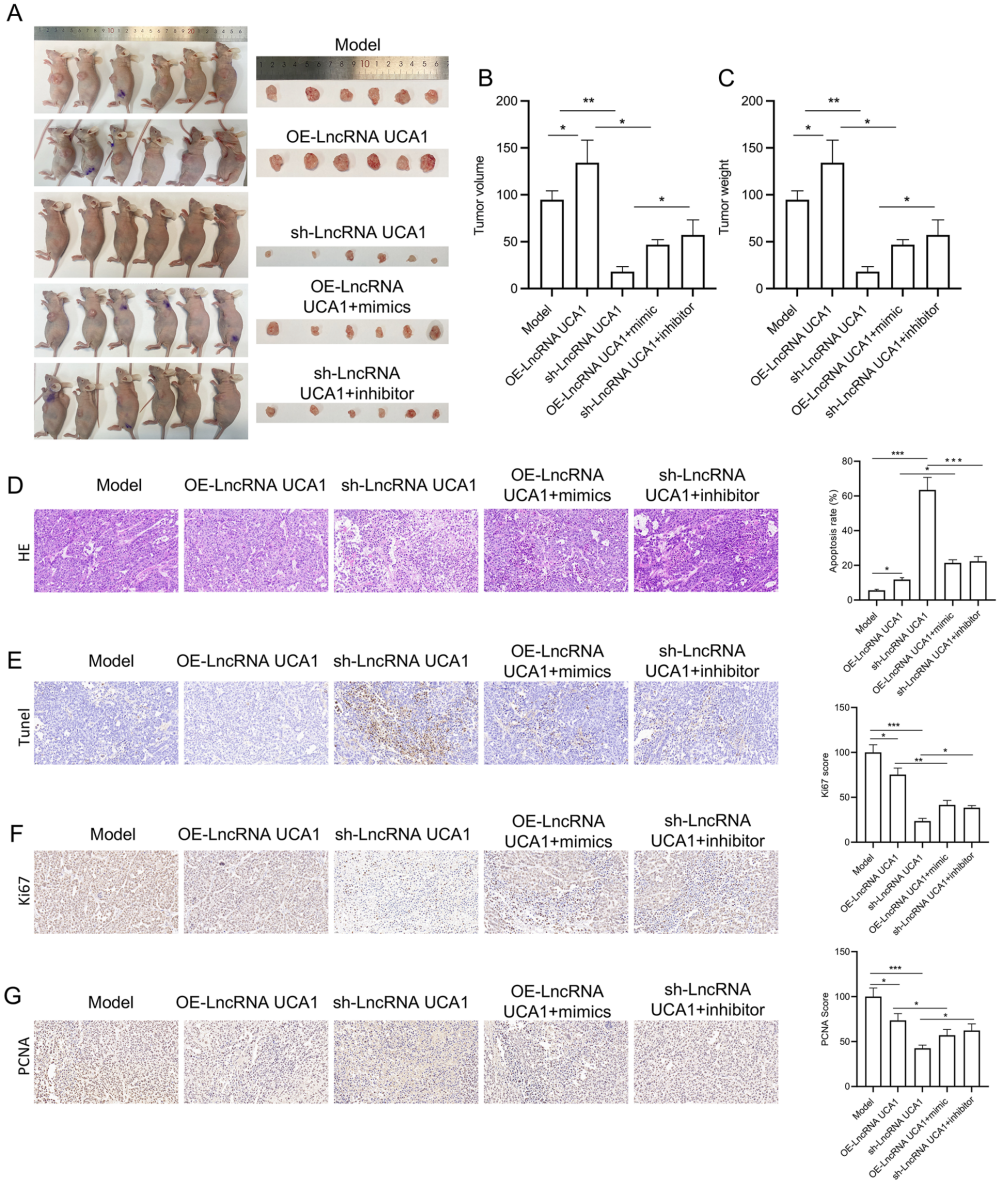
Figure 10 UCA1 overexpression is carcinogenic in a nude mouse tumor model by targeting miR-195-5p A mouse model of cervical cancer xenograft was constructed using the transduction of DDP/HeLa cells via lentiviruses. (A) Schematic diagram showing the subcutaneous tumors in nude mice after subcutaneous injection for 4 weeks. (B) Tumor volume in each group. (C) Tumor weight in each group. (D) The pathological structure of the transplanted tumor was evaluated via H&E staining. (E) Apoptosis of the tumor tissue was evaluated via TUNEL staining. (F,G) IHC staining of PCNA (F) and Ki67 (G) in tumor tissues. *P < 0.05, **P < 0.01, ***P < 0.001.

A nude mouse xenograft model was constructed to verify the effects of UCA1 and miR-195-5p on proliferation and apoptosis *in vivo*. DDP/HeLa cells were infected with UCA1-overexpressing, lncRNA *UCA1*-knockdown, miR-195-5p-overexpressing, or *miR-195-5p*-knockdown lentiviruses for subcutaneous tumor implantation in nude mice. Four weeks after cell inoculation, the nude mice were sacrificed, and tumor tissue was collected. Macroscopic observation revealed that tumors in the UCA1-overexpressing group were larger than those in the model group, whereas tumors in the UCA1-silenced group were smaller. miR-195-5p overexpression attenuated tumor growth mediated by UCA1 overexpression, whereas miR-195-5p inhibition increased tumor size, which was reduced by lncRNA *UCA1* silencing ( Figure 10A). The data revealed that UCA1 overexpression increased tumor volume and weight in nude mice by inhibiting miR-195-5p; *UCA1* silencing decreased the tumor volume and weight of nude mice by increasing miR-195-5p expression ( Figure 10B,C). The H&E staining results indicated that the cells in each group were irregular in shape and size, with some tumor tissues showing necrosis. The cancer cells were uniformly distributed and large, with large nuclei and marked atypia; the cytoplasm was clear, and eosinophilic staining was light, with no obvious fibrous stroma and an increased nucleoplasm ratio. Tumor cell growth was active, which is consistent with the characteristics of cervical cancer. Moreover, UCA1 overexpression attenuated apoptosis and necrosis, which was reversed by miR-195-5p overexpression; *UCA1* silencing enhanced apoptosis and necrosis, which was reversed by miR-195-5p inhibition ( Figure 10D). TUNEL staining revealed that UCA1 overexpression reduced the number of apoptotic cells in tumor tissues by inhibiting miR-195-5p, whereas *UCA1* silencing increased the number of apoptotic cells in tumor tissues by upregulating miR-195-5p ( Figure 10E). IHC staining data revealed that miR-195-5p overexpression markedly attenuated the stimulatory effect of UCA1 overexpression on the PCNA and Ki67 proteins, whereas miR-195-5p inhibition reversed the effect of *UCA1* silencing on reducing the levels of PCNA and Ki67 proteins ( Figure 10F,G).

### UCA1 overexpression upregulates IKBKB, BNIP3Δex2, and p-p65 and downregulates BNIP3 by targeting miR-195-5p in the tumor tissues of nude mice

**Figure 11:**
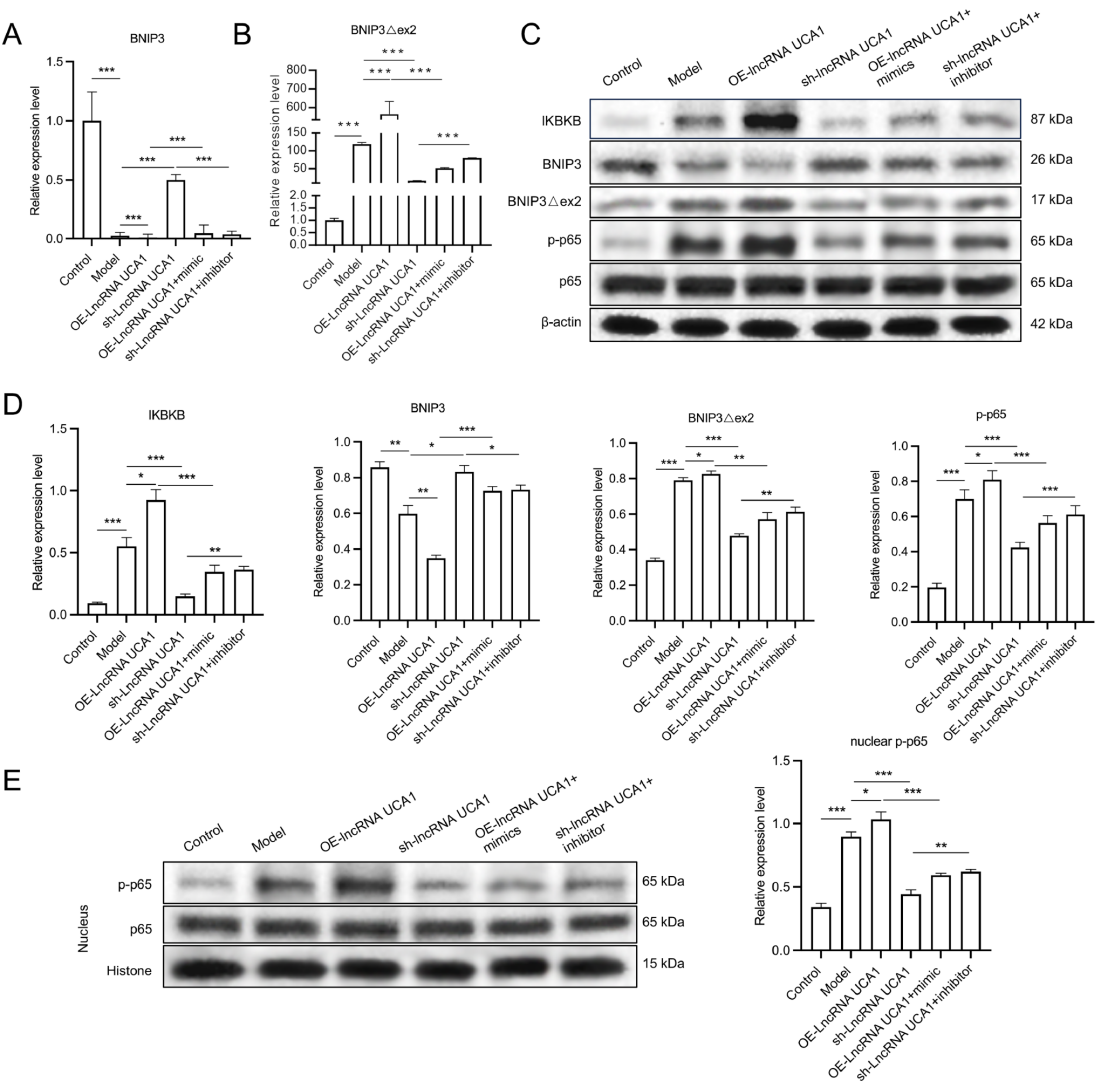
Figure 11 UCA1 overexpression upregulates IKBKB, BNIP3Δex2, and p-p65 expressions and downregulates BNIP3 expression by targeting miR-195-5p in the tumor tissues of nude mice (A,B) BNIP3 (A) and BNIP3Δex2 (B) expression levels were evaluated by qRT-PCR in the tumor tissues of nude mice. (C) Western blot analysis of IKBKB, BNIP3, BNIP3Δex2, p65, and p-p65 expressions in the tumor tissues of each group of nude mice. (D) Quantitative analysis of protein gray values. (E) Western blot analysis of p65 and p-p65 expressions in the nucleus. *P < 0.05, **P < 0.01, ***P < 0.001.

Additionally, qRT-PCR data revealed that the expression of BNIP3Δex2 in the tumor tissues of the model group was higher than that in the control group, whereas the BNIP3 expression in the model group was lower than that in the control group. UCA1 overexpression increased *BNIP3Δex2* mRNA level and decreased *BNIP3* mRNA level in the tumor tissues of nude mice. *UCA1* silencing decreased *BNIP3Δex2* mRNA level and increased *BNIP3* mRNA level. The overexpression of miR-195-5p partially reversed the increase in BNIP3Δex2 expression and the decrease in BNIP3 *e*xpression caused by UCA1 overexpression. miR-195-5p inhibition significantly attenuated the BNIP3 upregulation and BNIP3Δex2 reduction mediated by *UCA1* silencing ( Figure 11A,B). The western blot analysis results indicated that the IKBKB, BNIP3Δex2, and p-p65 proteins were upregulated, whereas the BNIP3 protein was downregulated in the model group compared with those in the control group. UCA1 overexpression notably upregulated IKBKB, BNIP3Δex2, and p-p65 expressions and downregulated BNIP3 expression in tumor tissues. *UCA1* silencing prominently downregulated IKBKB, BNIP3Δex2, and p-p65 expressions and upregulated BNIP3 expression in tumor tissues. Overexpression of miR-195-5p reversed the expression changes mediated by UCA1 overexpression. miR-195-5p inhibition also reversed the changes in protein expression mediated by *UCA1* silencing ( Figure 11C,D). Furthermore, UCA1 overexpression significantly increased p-p65 protein level in the nucleus by inhibiting miR-195-5p; *UCA1* silencing downregulated p-p65 protein level in the nucleus by increasing miR-195-5p level in tumor tissues ( Figure 11E).

## Discussion

An increasing number of lncRNAs are aberrantly expressed in cancer cells and play crucial roles as oncogenes or tumor suppressors [ 26, 27]. *UCA1* is located on chromosome 19p13.12 and is associated with multiple cancer processes [28]. It is also relevant to resistance to multiple chemotherapeutic agents, including cisplatin [29]. Our study demonstrated that UCA1 is highly expressed in DDP-resistant cervical cancer tissues and HeLa/DDP cells. Additionally, UCA1 overexpression accelerated proliferation, migration, and invasion while preventing apoptosis in HeLa/DPP cells; *UCA1* silencing had the opposite effect. Previous research has shown that UCA1 can increase the proliferation and invasion of cervical cancer cells via miR-204 [30], miR-299-3p [31], or miR-145 [19]. UCA1 prevents the survival and EMT of cervical cancer cells via miR-155 [32]. UCA1 could also enhance DDP resistance in cervical cancer [33]. Therefore, our study aligns with previous findings, showing that UCA1 is highly expressed in cisplatin-resistant cervical cancer patient tissues and cells *in vitro*; silencing of *UCA1* inhibits cervical cancer cell resistance to cisplatin and the malignant progression of cisplatin-resistant cervical cancer. However, this study did not elucidate the detailed mechanism by which UCA1 regulates cisplatin resistance, which needs further exploration. Additionally, only HeLa/DPP cell lines were used in this study, and future studies should include other DDP-resistant cervical cancer cell lines to confirm this conclusion.

LncRNAs can target miRNAs as ceRNAs, regulating the expressions and functions of their target genes in tumors [ 34, 35]. To reveal the regulatory role of UCA1 in cervical cancer DDP resistance and related mechanisms, our study predicted through bioinformatics that miRNAs, including miR-195-5p, might be regulated by UCA1. A literature review indicated that miR-195-5p is associated with cisplatin resistance in cancer. For example, miR-195-5p can reduce cisplatin resistance in ovarian cancer [36]; secretory clusterin can promote chemotherapy resistance in gastric cancer cells via miR-195-5p [37]; miR-195-5p can reverse chemoresistance in non-small cell lung cancer to cisplatin [38]; and miR-195-5p can attenuate cisplatin resistance in lung adenocarcinoma by affecting DNA damage [39]. Several studies have also shown that miR-195-5p can suppress proliferation and metastasis in cervical cancer via YAP1 [40] or ARL2 [41]; moreover, miR-195-5p can promote the apoptosis of cervical cancer cells [42]. These studies confirmed that miR-195-5p significantly inhibits cervical cancer progression. Therefore, we selected miR-195-5p as a target miRNA of UCA1. In the present study, miR-195-5p was validated as a direct target miRNA for UCA1 via a dual-luciferase reporter gene assay. Our study further revealed that UCA1 overexpression accelerates proliferation and metastasis while attenuating the apoptosis of HeLa/DPP cells by targeting the miR-195-5p/IKBKB axis. *In vivo*, UCA1 attenuates growth and accelerates apoptosis in DDP-resistant xenograft tumors by targeting miR-195-5p. Therefore, our data confirmed that *UCA1* silencing could attenuate DDP-resistant cervical cancer progression by regulating miR-195-5p.

IKBKB can form an IKK complex with IKKα and IKKγ, which play crucial roles in NF-κB activation [43]. Phosphorylation of the IKK complex can activate NF-κB and promote NF-κB nuclear translocation, regulating the transcription of a series of genes [44]. Studies have confirmed that IKBKB overexpression activates NF-κB, regulates apoptosis-related gene transcription, and attenuates apoptosis [ 22, 45]. Activation of the NF-κB pathway can also lead to DDP resistance in cervical cancer cells [23]. Thus, we speculated that UCA1 upregulates IKBKB to promote NF-κB activation, resulting in DDP resistance in cervical cancer. Our current study revealed that UCA1 could upregulate NF-κB (p-p65) and accelerate NF-κB nuclear transport in HeLa/DDP cells and that miR-195-5p overexpression could downregulate p-p65 and attenuate NF-κB nuclear transport in HeLa/DDP cells. Additionally, UCA1 could also increase p-p65 expression and induce NF-κB nuclear transport in transplanted tumors of nude mice by targeting miR-195-5p. Thus, our data confirmed that UCA1 enhanced NF-κB nuclear translocation via miR-195-5p in DDP-resistant cervical cancer. We also demonstrated that IKBKB overexpression increased p-p65 level in HeLa/DDP cells. However, whether UCA1 can affect the progression of DDP-resistant cervical cancer by regulating NF-κB nuclear translocation needs to be verified by further experiments, which is also an important direction for our future research.

BNIP3 is a member of the Bcl-2 protein family with proapoptotic effects [46]. BNIP3, a cisplatin regulator, is crucial for preventing tumor progression [47]. BNIP3 expression was reported to be absent in 18% of cervical cancer patients and decreased in 32% of cervical cancer patients [48]. Abnormal expression of BNIP3 is also associated with tumor chemoresistance [49]. NF-κB activation can downregulate BNIP3 to accelerate the malignancy of thyroid carcinoma [50]. In our study, we demonstrated that BNIP3 was downregulated in DDP-resistant cervical cancer. BNIP3 overexpression could suppress proliferation and invasion and accelerate the apoptosis of HeLa/DPP cells, and the effect of *BNIP3* silencing on the function of HeLa/DPP cells was opposite to that of BNIP3 overexpression. Moreover, BNIP3 could be downregulated by UCA1 and upregulated by miR-195-5p in HeLa/DPP cells; IKBKB overexpression increased BNIP3Δex2 level and decreased BNIP3 level in HeLa/DDP cells; UCA1 also downregulated BNIP3 by targeting miR-195-5p in transplanted tumors. However, it is unclear whether NF-κB can affect the malignant progression of cisplatin-resistant cervical cancer cells by downregulating BNIP3. In future studies, we will further explore this hypothesis: UCA1 might promote NF-κB nuclear translocation to downregulate BNIP3 in DDP-resistant cervical cancer via miR-195-5p/IKBKB.

BNIP3 alternative splicing affects tumor cell apoptosis and drug resistance [51]. BNIP3 transcription is regulated by a variety of transcription factors, which not only regulate the transcription level of BNIP3 but also lead to alternative splicing of BNIP3 [ 24, 52]. BNIP3 can be alternatively spliced to produce BNIP3Δex2 or BNIP3Δex3 in humans or mice. Neither splice variant contains a BH3-like domain or the key C-terminal transmembrane domain that is responsible for inducing cell death and mitochondria localization. BNIP3Δex2/BNIP3Δex3 can bind to BNIP3FL (BNIP3 full-length) and inhibit its mitochondrial localization, thereby inhibiting its proapoptotic function [53]. Another study revealed that different variants of BNIP3 have different C-termini and inhibit p53-induced mitochondrial disorders, autophagy, and apoptosis [54]. BNIP3Δex2 has been proven to have an antiapoptotic effect [25]. NF-κB can also promote BNIP3Δex2 expression during cellular hypoxia [25]. These results suggest that NF-κB might upregulate BNIP3Δex2 to attenuate cancer cell apoptosis. Our study demonstrated that BNIP3Δex2 was upregulated in DDP-resistant cervical cancer. BNIP3Δex2 overexpression accelerated the malignant progression of DDP-resistant cervical cancer cells, whereas *BNIP3Δex2* silencing had the opposite effect. UCA1 also upregulated BNIP3Δex2 via miR-195-5p in DDP-resistant cervical cancer. However, it is unclear whether UCA1 can accelerate NF-κB nuclear translocation by regulating IKBKB. In addition, whether NF-κB activation can enhance alternative BNIP3 splicing to increase BNIP3Δex2 production has not been demonstrated. It is also unclear whether IKBKB can promote BNIP3Δex2 production to accelerate DDP resistance in cervical cancer by activating NF-κB. These questions need to be addressed by rescue experiments in future research.

In conclusion, we preliminarily demonstrated that UCA1 accelerates the progression of DDP-resistant cervical cancer via the miR-195-5p/IKBKB axis. Additionally, UCA1 overexpression upregulated IKBKB, BNIP3Δex2, and p-p65 expressions, while downregulating BNIP3 expression by targeting miR-195-5p in cervical cancer. Therefore, this study provides a theoretical basis for treating DDP-resistant cervical cancer with the UCA1/miR-195-5p/IKBKB axis.

## Supporting information

24438Supplemental_Figures

## Supplementary Data

Supplementary data is available at *Acta Biochimica et Biophysica Sinica* online.
