# Supplementary material for: Long noncoding RNA UCA1 knockdown inhibits cisplatin-resistant cervical cancer tumorigenesis via the miR-195-5p/IKBKB axis: LncRNA UCA1 promotes cisplatin-resistant cervical cancer progression
Source: Acta Biochim Biophys Sin (Shanghai). 2025 Apr 22;57(9):1492–506. doi: 10.3724/abbs.2025032 (PMC12536463; doi:10.3724/abbs.2025032)
Supplement: 24438Supplemental_Figures [file 24438Supplemental_Figures.docx]

**
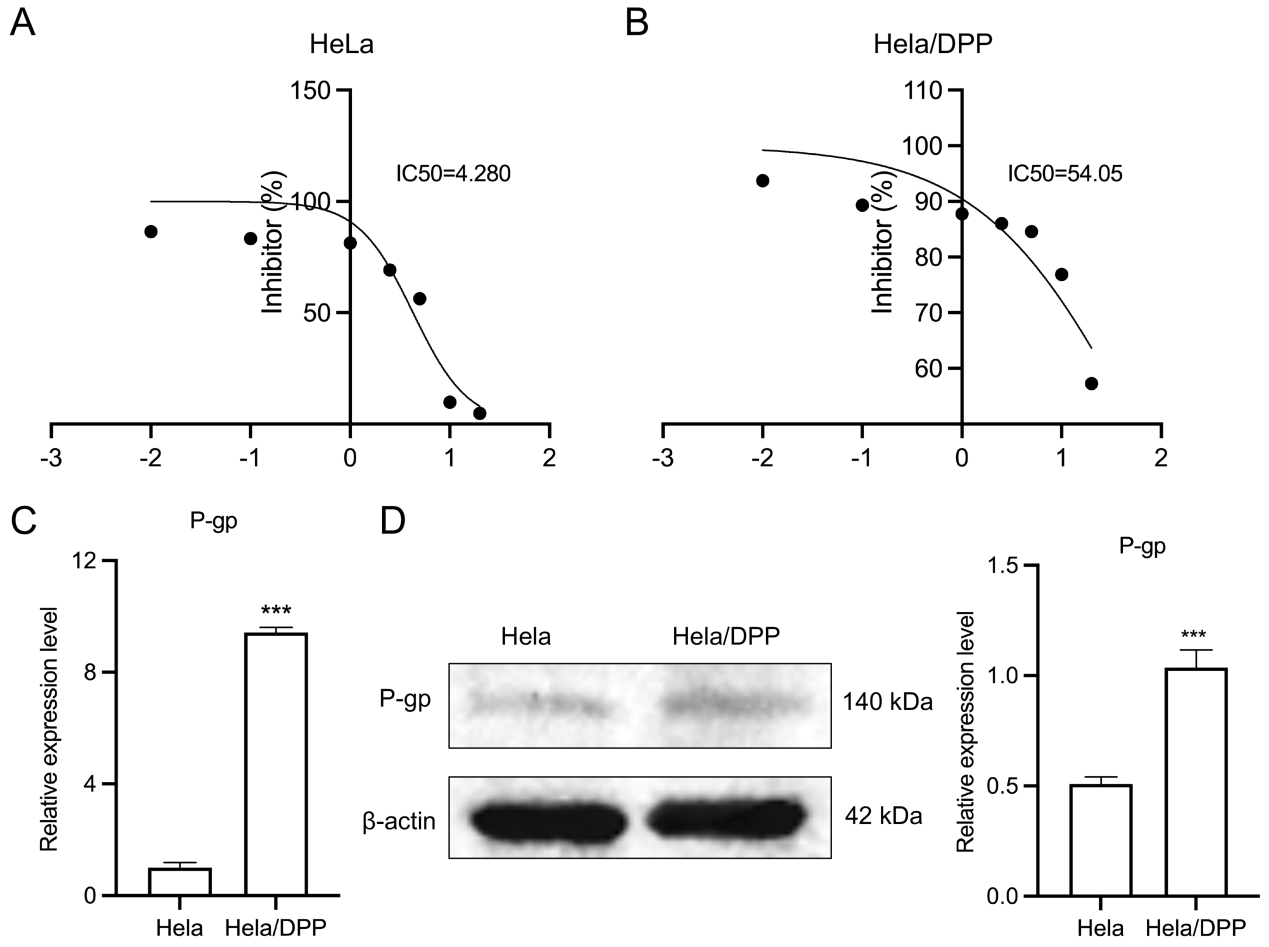
**

**Supplementary Figure S1. Generation and validation of DDP-resistant HeLa cells** DDP-resistant HeLa cells were generated with different concentrations of DDP. (A,B) CCK-8 assays revealed changes in the viability of HeLa and HeLa/DDP cells. (C) qRT-PCR assay for evaluating *p-gp* and *MDR1* levels in HeLa and HeLa/DDP cells. (D) P-gp protein expression was determined by western blot analysis. ****P* <0.001.


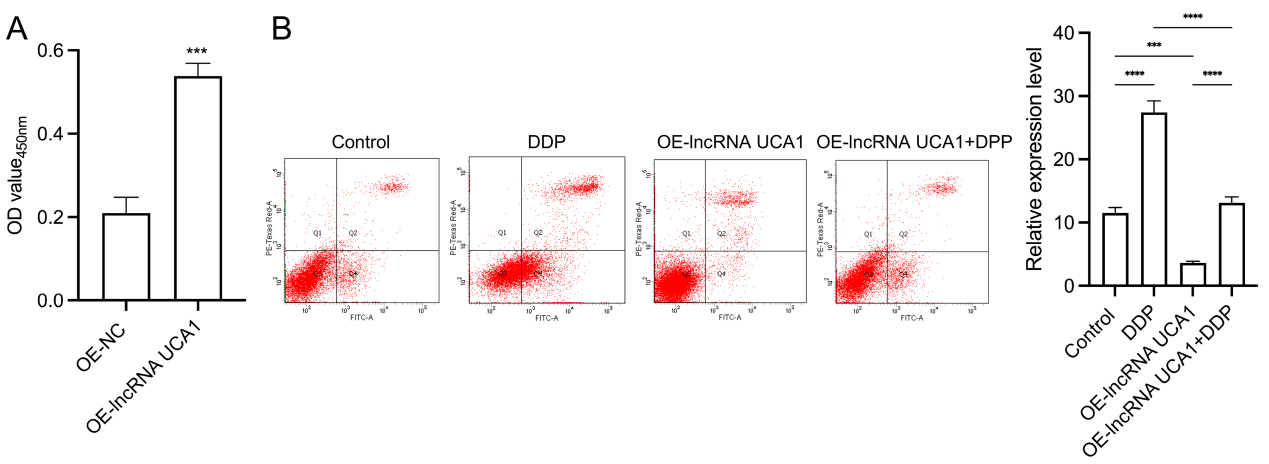


**Supplementary Figure S2. Effect of UCA1 overexpression on the proliferation and apoptosis of HeLa/DDP cells**  HeLa cells were transfected with the *UCA1* overexpression plasmid and treated with DDP. (A) CCK-8 assay of cell viability. (B) Flow cytometry assay of apoptosis. ***P* < 0.01, ****P* < 0.001.


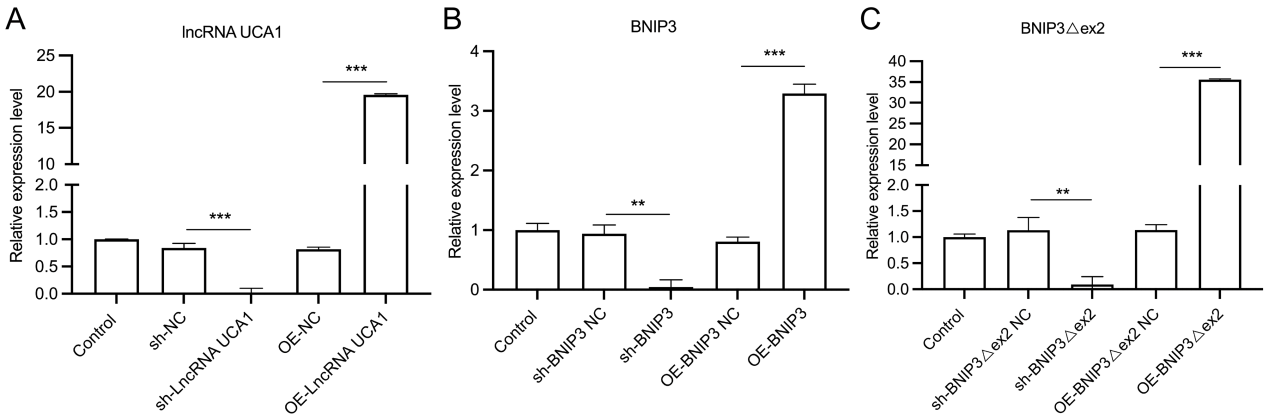


**Supplementary Figure S3.** **Validation of UCA1, BNIP3△ex2, and BNIP3 overexpressions and knockdown in HeLa/DDP cells** (A) qRT-PCR analysis of UCA1 expression in HeLa/DDP cells after *UCA1* silencing or overexpression. (B) After transfection with *BNIP3△ex2* shRNAs or overexpression plasmids, *BNIP3△ex2* mRNA was analyzed using qRT-PCR in HeLa/DDP cells. (C) qRT-PCR analysis of the effects of *BNIP3* shRNAs or overexpression plasmids in HeLa/DDP cells. ***P* < 0.01, ****P* < 0.001.
